# Overexpression of NMNAT3 improves mitochondrial function and enhances antioxidative stress capacity of bone marrow mesenchymal stem cells via the NAD^+^-Sirt3 pathway

**DOI:** 10.1042/BSR20211005

**Published:** 2022-01-14

**Authors:** Tao Wang, Fei Zhang, Wuxun Peng, Lei Wang, Jian Zhang, Wentao Dong, Xiaobin Tian, Chuan Ye, Yanlin Li, Yuekun Gong

**Affiliations:** 1 School of Clinical Medicine, Guizhou Medical University, Guiyang, Guizhou 550004, China; 2 Department of Traumatology, The Affliated Hospital of Guizhou Medical University, Guiyang, Guizhou 550004, China; 3 Department of Statistics, Guizhou Maternal and Child Health Hospital, Guiyang, Guizhou 550004, China; 4 Department of Orthopaedics, The Affliated Hospital of Guizhou Medical University, Guiyang, Guizhou 550004, China; 5 Department of Orthopaedics, The First Affiliated Hospital of Kunming Medical University, Kunming, Yunnan 650032, China

**Keywords:** apoptosis, mitochondrial dysfunction, oxidative stress, stem cells

## Abstract

Oxidative stress damage is a common problem in bone marrow mesenchymal stem cell (BMSC) transplantation. Under stress conditions, the mitochondrial function of BMSCs is disrupted, which accelerates senescence and apoptosis of BMSCs, ultimately leading to poor efficacy. Therefore, improving mitochondrial function and enhancing the antioxidative stress capacity of BMSCs may be an effective way of improving the survival rate and curative effect of BMSCs. In the present study, we have confirmed that overexpression of nicotinamide mononucleotide adenylyl transferase 3 (NMNAT3) improves mitochondrial function and resistance to stress-induced apoptosis in BMSCs. We further revealed the mechanism of NMNAT3-mediated resistance to stress-induced apoptosis in BMSCs. We increased the level of nicotinamide adenine dinucleotide (NAD^+^) by overexpressing NMNAT3 in BMSCs and found that it could significantly increase the activity of silent mating type information regulation 2 homolog 3 (Sirt3) and significantly decrease the acetylation levels of Sirt3-dependent deacetylation-related proteins isocitrate dehydrogenase 2 (Idh2) and Forkhead-box protein O3a (FOXO3a). These findings show that NMNAT3 may increase the activity of Sirt3 by increasing NAD^+^ levels. Our results confirm that the NMNAT3-NAD^+^-Sirt3 axis is a potential mechanism for improving mitochondrial function and enhancing antioxidative stress capacity of BMSCs. In the present study, we take advantage of the role of NMNAT3 in inhibiting stress-induced apoptosis of BMSCs and provide new methods and ideas for breaking through the bottleneck of transplantation efficacy of BMSCs in the clinic.

## Background

The survival of bone marrow mesenchymal stem cell (BMSC) transplantation has been the focus of current research investigations and has hindered clinical treatment [1–3]. Studies have shown that oxidative stress damage is mostly caused by the microenvironment, such as ischemia, hypoxia, and inflammation in the transplantation area, which seriously affects mitochondrial function and limits the proliferation, differentiation, and survival of implanted BMSCs. It accelerates senescence and apoptosis of BMSCs, which lead to poor curative effect *in vivo* [4–7]. Therefore, improving the mitochondrial function of BMSCs in the transplantation area and enhancing its antioxidative stress capacity may be an effective way to improve the survival rate and curative effect of BMSCs [8–11].

In recent years, studies related to mitochondrial function have shown that nicotinamide adenine dinucleotide (NAD^+^) is a key regulator of mitochondria. Intracellular NAD^+^ levels directly regulate important biological processes, such as cell rhythm, senescence, resistance, and cytoprotection [12–14]. This is mainly related to a variety of NAD^+^-dependent enzymes that are involved in important cellular biological reactions, including protein deacetylation reactions related to stress and senescence, polyadenylate diphosphate ribose reactions during DNA repair, and adenosine diphosphate ribose cyclization reactions that regulate calcium channels [15–18]. Among these, deacetylation modification of mitochondrial proteins plays an important role in regulating mitochondrial function and in adapting to stressors [19,20]. Deacetylation modification of mitochondrial proteins is mainly regulated by silent mating type information regulation 2 homolog 3 (Sirt3) of the sirtuin gene family [21–24]. The deacetylase activity of Sirt3 depends on NAD^+^ activation. Depletion of intracellular NAD^+^ content can lead to a decrease in Sirt3 activity, which directly affects the stability of energy metabolism and balance of the antioxidant defense system in mitochondria [25–28]. Therefore, interfering with the NAD^+^ homeostasis and then improving the activity of Sirt3 are important means to improve mitochondrial dysfunction [29].

The regulation of NAD^+^ synthesis involves many enzymes, including nicotinamide mononucleotide adenylyl transferase 3 (NMNAT3), which plays an essential role in the synthesis of NAD^+^ in the mitochondria [30–32]. Overexpression of NMNAT3 can significantly increase the levels of NAD^+^ in the mitochondria of aged mouse tail-tip fibroblasts and human umbilical cord blood mesenchymal stem cells [33]. Similarly, NMNAT3-overexpression mice can increase NAD^+^ levels in the mitochondria, improve mitochondrial biosynthesis and energy metabolism, and restore mitochondrial function [34]. However, whether NMNAT3 can enhance the antioxidative stress capacity of BMSCs and improve the survival rate of BMSCs under oxidative stress remain unclear.

In the present study, we overexpressed the *NMNAT3* gene in BMSCs *in vitro*. We simulated the microenvironment of oxidative stress in the transplantation area by using hydrogen peroxide (H_2_O_2_) to evaluate the effects of NMNAT3 on the mitochondrial function and antioxidative stress capacity of BMSCs under oxidative stress [35]. We also present the possible regulatory mechanism of NMNAT3, provide a new method for improving the survival of stem cell during transplantation, promote the clinical transformation of stem cell, and open up novel ideas for treatment.

## Materials and methods

### Animals

A total of 20 young New Zealand white rabbits (average weight: 2.0 ± 0.5 kg) were provided by the Laboratory Animal Center of Guizhou Medical University (Guiyang, Guizhou, China). All animal experiments took place at the Laboratory Animal Center of Guizhou Medical University. All the animal protocols were approved by the Experimental Animal Bioethics Committee of Guizhou Medical University (grant number 1900590), and all the procedures were conducted in strict accordance with the guidelines for the Care and Use of Experimental Animals issued by the National Institutes of Health (NIH Publication Number 85-23, 1996 revised). After the experiment, the rabbits were killed with pentobarbital (120 mg/kg) (Sigma–Aldrich, Darmstadt, Germany) under anesthesia. Finally, the animal carcasses were incinerated by the Animal Experimental Center of Guizhou Medical University.

### Cell culture

The rabbits were anesthetized with pentobarbital (40 mg/kg) by intravenous injection, then the rabbits were fixed on the operating table for small animals in the supine position and disinfected using standard procedures. The bone marrow was extracted from the distal femur and proximal tibia of rabbits by bone puncture, and then the rabbits were killed with pentobarbital (120 mg/kg) under anesthesia. Four to five milliliters of bone marrow fluid was extracted and, as mentioned earlier, cells were separated by density-gradient centrifugation [36,37]. Single cell precipitations were resuspended in 5 ml complete l-glutamine Dulbecco’s modified Eagle’s medium (l-DMEM) (Gibco, U.S.A.) containing 10% fetal bovine serum (FBS) (Gibco, U.S.A.) and 1% double antibody (HyClone, U.S.A.), and then transferred it into the 25-cm^2^ cell culture bottle (Corning, U.S.A.). The cells were cultured at 37°C and 5% CO_2_. When cell confluence reached 90%, the cells were digested with 1 ml of 0.25% trypsin in 0.02% ethylenediaminetetraacetic acid (EDTA) (Gibco, U.S.A.) at 37°C and passaged at a ratio of 1:3.

### Mitochondrial extraction

According to the experimental grouping, the treated BMSCs were washed with phosphate-buffered saline (PBS; HyClone, U.S.A.) two to three times; then the cells were digested with 1 ml EDTA at 37°C, and then centrifuged at 600×***g***/min for 5 min. According to the instructions provided in the Cell Mitochondrial Isolation Kit (Beyotime, China), 1.5 ml of the mitochondrial separation reagent was mixed with the protease inhibitor PMSF to resuspend the cells, placed in an ice bath for 15 min, and then the cell suspension was transferred into a glass homogenizer and ground 30 times. The cell homogenate was obtained and centrifuged at 600×***g***/min for 10 min at 4°C, the supernatant was transferred to another centrifuge tube, the supernatant was centrifuged at 11000×***g***/min for 10 min at 4°C, and the supernatant was removed. The precipitate consisted of mitochondria and was immediately used or resuspended in 1 ml of mitochondrial storage solution and stored at −80°C.

### BMSC surface antigen identification

Third-generation BMSCs were collected and resuspended in complete l-DMEM, and the cell concentration was adjusted to 2.0 × 10^7^ cells/ml. Approximately 50 μl of the cell suspension was placed into the flow tube (Corning, U.S.A.), followed by 5 μl of anti-CD29/AF647, anti-CD90/PE-Cy™7, anti-CD106/PE, anti-CD45/FITC, and anti-CD11b/V450 (BD, U.S.A.). Then, 45 µl of buffer (HyClone, U.S.A.) was added to each tube. The tubes were incubated at room temperature for 30 min, washed twice with staining buffer, and then 500 µl of buffer was added to each tube for detection by flow cytometry (Beckman, U.S.A.).

### BMSC multidirectional differentiation induction

Third-generation rabbit BMSCs showing good growth were selected, and the cell concentration was adjusted to 2.0 × 10^4^ cells/cm^2^ using an osteogenic, adipogenic, and chondrogenic induction differentiation kit (Cyagen Biosciences, Suzhou, China). The cells were inoculated on a six-well plate using 2 ml of the cell suspension per well. At various degrees of cell confluence (60, 100%, or 60%), the culture medium of the experimental group was replaced by an induced differentiation medium, whereas that of the control group remained in complete l-DMEM. The BMSCs were identified by staining 2, 3, and 4 weeks after differentiation. ALP staining was used to identify osteogenic differentiation, Oil Red O staining was used to identify adipogenic differentiation, and Alcian Blue staining was used to identify chondrogenic differentiation.

### Lentivirus infection

Third-generation rabbit BMSCs were divided into three groups according to the transfection conditions: group A (BMSCs), group B (BMSCs+Lv-EGFP), and group C (BMSCs+Lv-NMNAT3-EGFP). Lentivirus transfection (HengYuan Biological Technology, Shanghai, China) (MOI = 100) was performed for 12 h according to the experimental group [37]. After 12 h, the culture medium was changed to complete l-DMEM. After 3 days, the expression of green fluorescent protein was observed under an inverted fluorescence microscope, and the transfection efficiency was calculated. At 5 days post-transfection, the cells were selected with puromycin (2 μg/ml) (Solarbio, Beijing, China) for 2 weeks to construct cell lines with stable knockdown or overexpression. The transfection efficiency was verified by quantitative real-time PCR (qPCR).

### qPCR

Total RNA was extracted from cells using TRIzol (Invitrogen, U.S.A.). Then, 10 μl of the extracted RNA was reverse-transcribed using RevertAid™ First-Strand cDNA Synthesis Kit (Sangon Biotech, China). The reaction parameters were as follows: 65°C for 5 min, 42°C for 30 min, and 70°C for 10 min. One microliter of the resulting cDNA was used for qPCR using the following cycle parameters: 40 cycles at 95°C for 3 min, 95°C for 3 min, and 60°C for 30 s. The housekeeping gene *β-actin* was used as internal reference. Using the comparative threshold method, the relative expression quantity of the target gene = 2^−ΔΔ*C*_t_^ was used to determine the initial amount of template of the sample. The sequences of the rabbit primers (Sangon Biotech, China) were as follows: NMNAT3-F: 5′-CCCGTCAATGACAGCTACAGGAAG-3′; NMNAT3-R: 5′-AGCACCTTCACCGTCTCCATCC-3′; β-actin-F: 5′-TCCCTGGAGAAGAGCTACGA-3′; β-actin-R: 5′-GTACAGGT CCTTGCGGATGT-3′.

### Immunoblotting and immunoprecipitation

Total protein was extracted from each group by cell lysis (Beyotime, China), and total protein concentration was determined using a BCA protein concentration detection kit (Solarbio, Beijing, China). Equal amounts of proteins obtained from different kinds of cell lysates were separated by 10 or 15% SDS/PAGE (Beyotime, China), transferred on to PVDF membranes, and subjected to Western blotting using an ECL chemiluminescence reagent (Merck Millipore). β-actin and COX IV proteins were used as internal references, and the relative expression of the target protein was calculated according to the gray value of the protein band/β-actin (or COX IV) protein band gray value. For co-immunoprecipitation, we incubated the cytosolic fractions with antibodies for 12 h at 4°C followed by Protein A/G Plus-Agarose (Sigma–Aldrich, Darmstadt, Germany) for 3 h at 4°C on a rotating device. Immunoprecipitates were collected by centrifugation at 6000×***g***/min at 4°C and washed with lysis buffer (20 mM Tris pH 7.5, 150 mM NaCl, 1 mM EDTA, 1% Triton X-100, proteases, and phosphatase inhibitors; Sigma–Aldrich). We eluted the pellets by heating these at 95°C for 5 min in electrophoresis sample buffer. Then, Western blot detection was conducted according to the above method. The primary antibodies; anti-NMNAT3, anti-PGC-1α, anti-NRF1, anti-Sirt3, anti-isocitrate dehydrogenase 2 (Idh2), and anti-Forkhead-box protein O3a (FOXO3a; diluted by 1:300) and internal reference antibodies; anti-β-actin and anti-COX IV (diluted by 1:7500) were purchased from Abcam (Cambridge, U.S.A.).

### Cellular oxidative stress model

The BMSCs of each experimental group were treated with H_2_O_2_ (Chengdu Jinshan Chemical Reagent Co., Ltd.) at a concentration of 600 μM for 24 h [35], whereas the control group remained in culture using complete l-DMEM.

### Ultrastructural assessment of mitochondria

The BMSCs of each group were digested, centrifuged, and collected into EP tubes and fixed with 3% glutaraldehyde (Solarbio, Beijing, China) and 1% osmic acid (Solarbio, Beijing, China). Then, the cells were dehydrated across an ethanol (Solarbio, Beijing, China) gradient (50, 70, 80, 90, and 95%) for 15 min each, then in 100% ethanol for 20 min, and finally with pure acetone (Solarbio, Beijing, China) for 20 min. Then, these were placed in pure acetone and embedding solution (Beyotime, China) (2:1) at room temperature for 4 h, pure acetone and embedding solution (1:2) at room temperature overnight, and pure embedding solution at 37°C for 3 h. The samples were placed in an oven at 37°C overnight, 45°C for 12 h, and 60°C for 24 h. Finally, an ultrathin microtome (Leica, Germany) was used to prepare 50–60-nm ultrathin sections and stained with 3% uranyl acetate-lead citrate (Gibco, U.S.A.). The ultrastructural changes in the mitochondria were observed under a transmission electron microscope (Hitachi, Tokyo, Japan).

### Mitochondrial membrane potential

After oxidative stress treatment to BMSCs, the cells in each group were washed with PBS twice, and the reaction mixture was prepared according to the instructions of the Mitochondrial Membrane Potential Assay Kit with TMRE (Beyotime, China). The reaction mixture was added, incubated at 37°C for 30 min, washed thrice with PBS, and fluorescence was observed under a laser confocal microscope (Zeiss, Germany). The relative intensity of red fluorescence was calculated using an imaging software.

### Detection of adenosine triphosphate, NAD^+^, malondialdehyde, catalase, manganese superoxide dismutase, and reduced glutathione/oxidized glutathione using a multifunctional microplate reader

The BMSCs of each group were collected according to the experimental groups, and according to the instructions of the adenosine triphosphate (ATP) assay kit (Beyotime, China), NAD^+^/NADH assay kit with WST-8 (Beyotime, China), lipid peroxidation malondialdehyde (MDA) assay kit (Beyotime, China), catalase (CAT) assay kit (Beyotime, China), Cu/Zn-SOD and manganese superoxide dismutase (Mn-SOD) assay kit with WST-8 (Beyotime, China), and reduced glutathione/oxidized glutathione (GSH/GSSG) assay kit (Beyotime, China); the appropriate lysate was added to the samples of each group, and then centrifuged at 12000×***g***/min for 5 min at 4°C, then the supernatant was collected for further analysis. Standard and test solutions were prepared according to the instructions of the kit. Then, 20-μl test samples or standard samples were added successively to the 96-well plate, repeated for another three wells for each group, 100 μl of the detection working solution was added, mixed well, and incubated for 25–30 min at 37°C or room temperature. Finally, the absorbance at 532 nm (ATP assay kit), 520 nm (NAD^+^/NADH assay kit with WST-8), 532 nm (lipid peroxidation MDA assay kit), 520 nm (CAT assay kit), 450 nm (Cu/Zn-SOD and Mn-SOD assay kit with WST-8), and 412 nm (GSH and GSSG assay kit) were detected using a multifunctional microplate reader (Biotech, U.S.A.). Standard curves were drawn based on the absorbance value, and the concentration or activity of the sample was calculated using the standard curve.

### DHE staining

Each group of BMSCs was cultured in confocal Petri dishes, and when confluence reached 85%, the protocol provided in the reactive oxygen species (ROS) detection kit-DHE (Sigma) was performed. After the cells in each group were treated, changes in red fluorescence intensity was assessed using a confocal microscope (Leica SP5, Heidelberg, Germany).

### Senescence associated-β-galactosidase staining

The cells in the experimental group were fixed at room temperature for 15 min, and a staining solution was prepared according to the cell senescence β-galactosidase (SA-β-Gal) staining kit (Beyotime, China). After adding the working solution, the cells were cultured overnight at 37°C and then assessed using an ordinary optical microscope.

### Cell viability and proliferation

The density of BMSCs in each group was adjusted to (2–3) × 10^3^ cells/well and cultured in a 96-well plate. Four wells were used for each group, using a total of 12 plates, and further cultured at 37°C and with 5% CO_2_. Then, a plate was used at each time point following the instructions of the cell counting kit-8 (CCK-8) kit (Solarbio, Beijing, China). The cells of each group were treated with the working reagent of the kit, and then the absorbance (OD value) of the cells at a wavelength of 450 nm was determined using a microplate reader. The same method was used in assessing the 96-well plate each day. The cell growth of each group was presented as a graph with the *x*-axis representing the number of days in culture and the *y*-axis depicting the average absorbance value.

### TUNEL/DAPI detection of apoptosis

The treated cells were digested and centrifuged, and the cell density was adjusted to 2.5 × 10^4^/ml before inoculating in a confocal Petri dish. The cells of each group were treated strictly in accordance with the instructions of the terminal deoxynucleotidyl transferase dUTP nick-end labeling (TUNEL) and 4′,6-diamidino-2-phenylindole (DAPI) detection kits (TUNEL: Beyotime, China; DAPI: Solarbio, Beijing, China). The samples were observed under a confocal microscope (Zeiss, Germany) and images were collected.

### Determination of Sirt3 activity

After oxidative stress treatment of the BMSCs, the cells were lysed with cell lysis buffer, centrifuged at 12500×***g***/min for 5 min at 4°C, and the supernatant was collected. According to the instructions of the SIRT3 activity assay kit (MBL, Japan), the supernatant was mixed with the working solution provided in the SIRT3 activity assay kit, and then incubated in a 96-well plate at room temperature for 30 min. After that, the relative fluorescence intensity was detected using a microplate reader (excitation wavelength: 340 nm; emission wavelength: 460 nm), and the relative fluorescence intensity of the sample was used to reflect the enzyme activity of the sample.

### Statistical analysis

All the statistical data were calculated and graphed using the SPSS software package ver. 20.0 (SPSS, Inc., Chicago, IL, U.S.A.) and GraphPad Prism 6.0 (GraphPad software, La Jolla, CA, U.S.A.). Each experiment was performed at least in triplicate, and the data were presented as mean ± standard deviation. We used the Kolmogorov–Smirnov test to analyze the normality of the data. Statistical differences were analyzed by unpaired two-tailed Student’s *t* test for comparison between two groups and one-way ANOVA followed by Tukey’s post hoc test for multiple comparisons (three or more groups). Differences with *P*-values <0.05 were deemed to be statistically significant.

## Results

### Overexpression of NMNAT3 in BMSCs results in an increase in NAD^+^ levels

**Figure 1:**
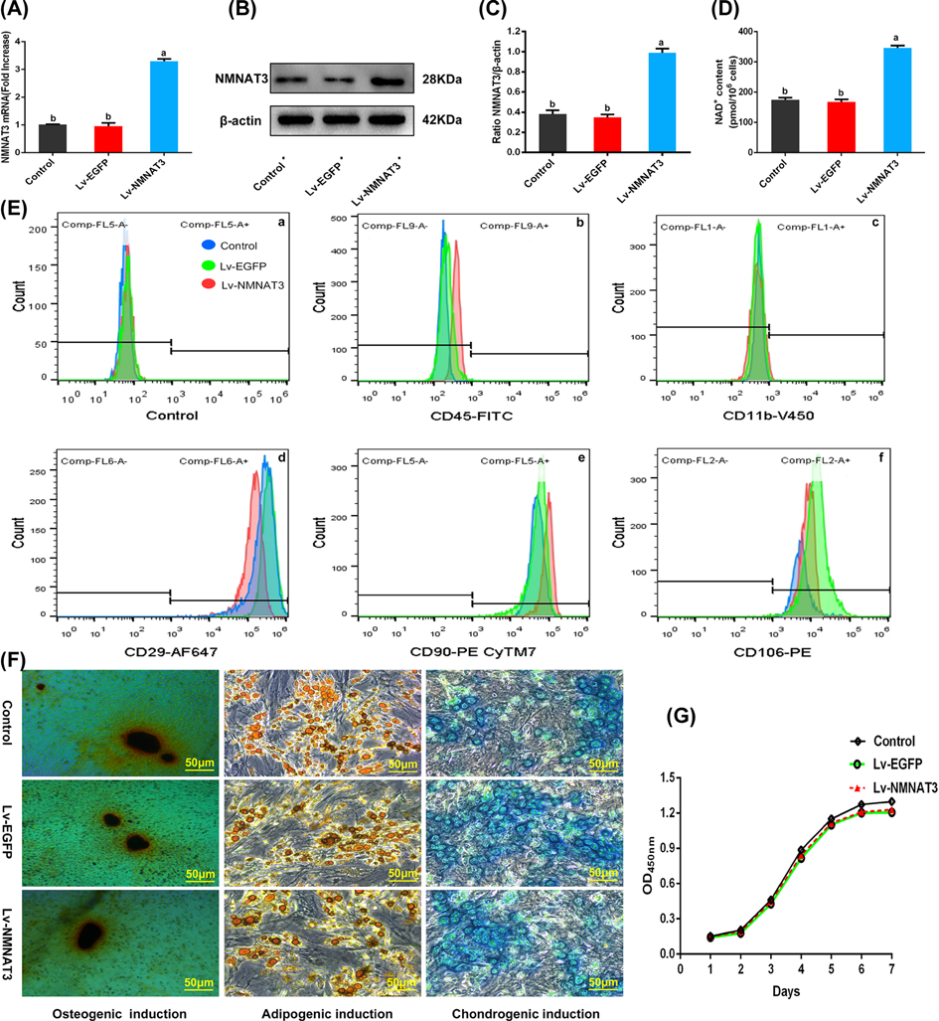
Expression of NMNAT3 and the function of BMSCs after transfection (**A**) qPCR analysis of relative expression of NMNAT3 mRNA (*n*=3), significant differences (*P*<0.05) were observed between a and b. (**B**,**C**) Western blot analysis of NMNAT3 protein expression (*n*=3), significant differences (*P*<0.05) were observed between a and b. (**D**) Detection of NAD^+^ levels in cells (*n*=3), significant differences (*P*<0.05) were observed between a and b. (**E**) Identification of BMSC surface antigen by flow cytometry (*n*=3). (**F**) Observation of staining induced by multidirectional differentiation (*n*=3). (**G**) Cell proliferation curve (*n*=4). All the experiments were repeated at least thrice (*n*≥3). Data were presented as means ± SD. *P*<0.05 by one-way ANOVA with Tukey’s post hoc test (three or more groups). Abbreviation: mRNA, messenger RNA.

NMNAT3 is a key enzyme in the synthesis of NAD^+^ in mitochondria, which plays an essential role in the regulation of NAD^+^ homeostasis [30–34]. Here, we inserted the CDS region of the *NMNAT3* gene into the overexpressed lentiviral vector (Lv). Then, the lentivirus was transfected into the BMSCs. PCR analysis showed that the mRNA level in the overexpression group (Lv-NMNAT3) was significantly higher than that in the control (Control) or the empty virus (Lv-EGFP) group (*P*<0.05) (Figure 1A), and Western blotting revealed that NMNAT3 protein expression in the Lv-NMNAT3 group was significantly higher than in the Control or the Lv-EGFP group (*P*<0.05) (Figure 1B,C, Supplementary Figure S1). These results confirmed the successful overexpression of the *NMNAT3* gene in BMSCs.

To further assess the effect of NMNAT3 overexpression on NAD^+^ levels in BMSCs, we detected the NAD^+^ levels in cells. The results showed that the NAD^+^ levels in BMSCs modified by the *NMNAT3* gene were significantly higher than the Control or Lv-EGFP group (*P*<0.05), and there was no significant difference between the Control and Lv-EGFP groups (Figure 1D).

### NMNAT3-overexpressing BMSCs retain its mesenchymal characteristics and proliferative ability

We successfully obtained a stable strain of BMSCs that overexpressed the *NMNAT3* gene; however, whether the *NMNAT3* gene affects the mesenchymal characteristics and proliferation of BMSCs remains unclear [38]. Flow cytometry showed that the BMSCs modified by NMNAT3 still exhibited high levels of typical mesenchymal stem cell markers CD29, CD90 and CD106 (>99%), while the levels of hematopoietic CD45 and monocyte CD11b markers were low (<1%). These expression profiles were similar to those of the Control BMSCs (Figure 1E and Table 1). To assess its differentiation ability [39], we used kits to induce osteogenesis, chondrogenesis, and adipogenic differentiation. The results were assessed by Alizarin Red, Alcian Blue, and Oil Red O staining (Figure 1F). In addition, we used a CCK-8 kit to determine the proliferation ability of BMSCs in each group. The growth curve showed that the trend of cell proliferation in each group was similar, with no significant differences. Each group underwent a latent adaptation period of 1–2 days, and then proliferated on the third day, entered the logarithmic growth phase, remained in the logarithmic growth phase for ∼3 days, and finally reached a plateau (Figure 1G).

**Table 1:** Analyzed CD markers of the BMSCs in control group (Control), empty virus group (Lv-EGFP), and NMNAT3 overexpression group (Lv-NMNAT3) by flow cytometry

Stem cell marker	Control	Lv-EGFP	Lv-NMNAT3
CD45	0.04%	0.07%^1^	0.06%^2^
CD11b	0.38%	0.59%^1^	0.67%^2^
CD29	99.97%	99.79%^1^	100.00%^2^
CD90	100.00%	99.99%^1^	100.00%^2^
CD106	99.72%	99.62%^1^	99.68%^2^

The expression of CD markers in BMSCs of each group, *n*=3. ^1^*P*>0.05 vs Control. ^2^*P*>0.05 vs Lv-EGFP.

The above data showed that gene modification does not change the mesenchymal characteristics of BMSCs, as these continued to exhibit high proliferation and differentiation rates.

### Protection of mitochondrial function of BMSCs after NMNAT3 overexpression under oxidative stress

**Figure 2:**
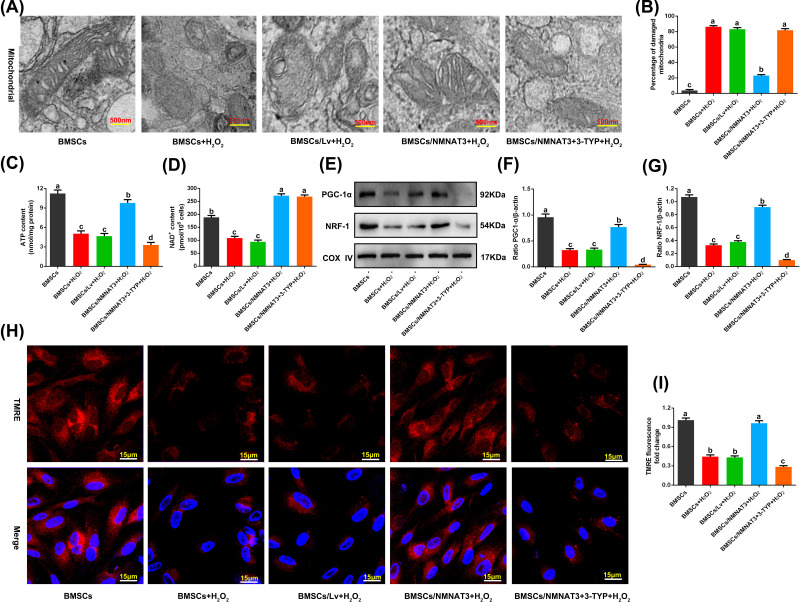
Mitochondrial structure and function of BMSCs under oxidative stress (**A**) Observation of ultrastructure of mitochondria by transmission electron microscopy (*n*=3). (**B**) The proportion of mitochondria with altered ultrastructure under different experimental conditions (*n*=3), significant differences (*P*<0.05) were observed among a–c. (**C**) Detection of intracellular ATP levels (*n*=3), significant differences (*P*<0.05) were observed among a–d. (**D**) Detection of intracellular NAD^+^ levels (*n*=3), significant differences (*P*<0.05) were observed among a–c. (**E**) Western blot analysis of PGC-1α and NRF1 expression in BMSCs (*n*=3). (**F**) Analysis of relative expression of PGC-1α (*n*=3), significant differences (*P*<0.05) were observed among a–d. (**G**) Analysis of relative expression of NRF1 (*n*=3), significant differences (*P*<0.05) were observed among a–d. (**H,I**) Detection of mitochondrial membrane potential using a mitochondrial membrane potential assay kit with TMRE (*n*=3). All the experiments were repeated at least thrice (*n*≥3), significant differences (*P*<0.05) were observed among a–c. Data were presented as means ± SD. *P*<0.05 by one-way ANOVA with Tukey’s post hoc test (three or more groups). Abbreviations: COX IV, cytochrome *c* oxidase IV; 3-TYP, 3-(^1^H-1,2,3-triazol-4-yl) pyridine.

Mitochondrial dysfunction in stem cells is caused by oxidative stress and is mainly responsible for stress apoptosis of stem cells [5,8–11]. BMSCs were treated with high concentrations of H_2_O_2_ (600 μM) for 24 h to simulate an oxidative stress microenvironment and construct a cell model of oxidative stress damage [35]. The effects of NMNAT3 on mitochondrial function of BMSCs under oxidative stress were studied by assessing mitochondrial ultrastructure, ATP content, NAD^+^ levels, regulators of mitochondrial biogenesis peroxisome proliferator-activated receptor γ coactivator-1α (PGC-1α) and nuclear respiratory factor 1 (NRF1), and mitochondrial membrane potential [40,41]. The results showed that the mitochondrial ultrastructure of BMSCs in H_2_O_2_-treated (BMSCs+H_2_O_2_ and BMSCs/Lv+H_2_O_2_) groups was destroyed, the mitochondrial crest had fused and broken (Figure 2A), and the proportion of mitochondria showing altered ultrastructure significantly increased (Figure 2B). Moreover, intracellular ATP and NAD^+^ levels decreased (Figure 2C,D), the expression of mitochondrial biogenesis regulatory factors PGC-1α and NRF1 were down-regulated (Figure 2E–G, Supplementary Figure S2), and the mitochondrial membrane potential decreased (Figure 2H,I). On the contrary, after overexpression of NMNAT3 in BMSCs (BMSCs/NMNAT3+H_2_O_2_), the changes in mitochondrial ultrastructure were restored, intracellular ATP and NAD^+^ levels increased, mitochondrial biogenesis regulatory factors PGC-1α and NRF1 were up-regulated, and mitochondrial membrane potential increased (Figure 2A–I). In summary, these results suggest that *NMNAT3* gene overexpression can effectively recover the damage of mitochondrial structure and functions of BMSCs under oxidative stress.

### NMNAT3 enhances the antioxidative stress capacity of BMSCs

**Figure 3:**
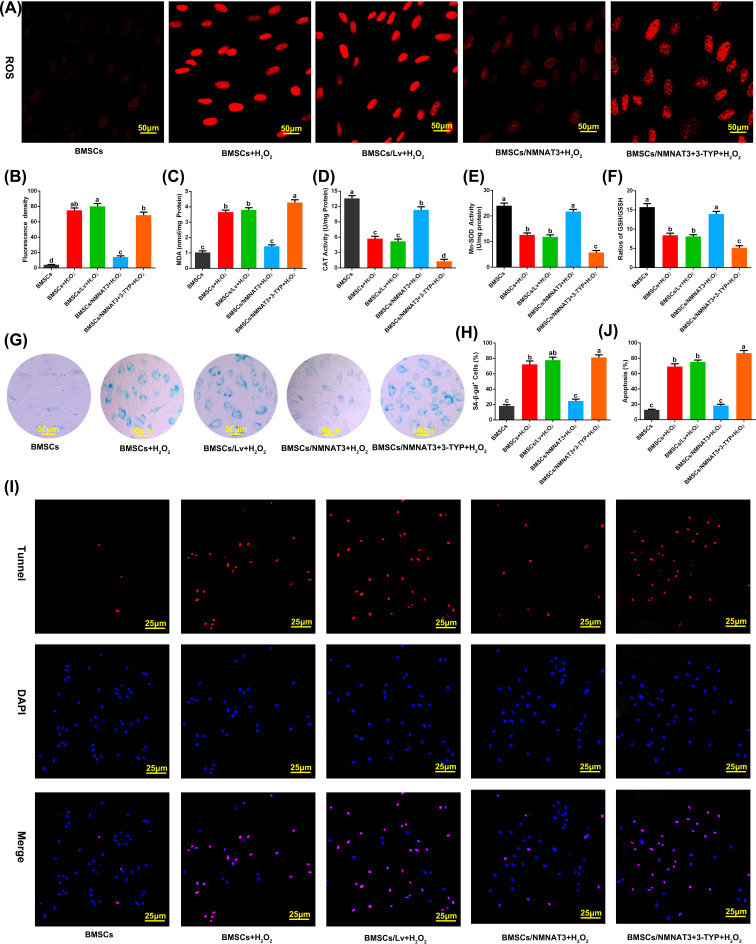
Antioxidative stress capacity of BMSCs under oxidative stress (**A,B**) DHE fluorescence probe detection of ROS (*n*=3), significant differences (*P*<0.05) were observed among a–d. (**C**) MDA in BMSCs content detection, significant differences (*P*<0.05) were observed among a–c. (**D**) Analysis of CAT activity in BMSCs, significant differences (*P*<0.05) were observed among a–d. (**E**) Analysis of Mn-SOD activity in BMSCs, significant differences (*P*<0.05) were observed among a–c. (**F**) Detection of the ratio of GSH/GSSH in BMSCs, significant differences (*P*<0.05) were observed among a–c. (**G,H**) Detection of β-gal activity by β-gal staining (*n*=4), significant differences (*P*<0.05) were observed among a–c. (**I,J**) TUNEL/DAPI staining method for detecting apoptosis (*n*=4), significant differences (*P*<0.05) were observed among a–c. All the experiments were repeated at least thrice (*n*≥3). Data were presented as means ± SD. *P*<0.05 by one-way ANOVA with Tukey’s post hoc test (three or more groups).

Overexpression of NMNAT3 significantly improved the mitochondrial function of BMSCs under oxidative stress. Subsequently, we further evaluated the antioxidative stress capacity of BMSCs modified by NMNAT3. We analyzed the basic antioxidative stress capacity of BMSCs, and we detected the levels of ROS and MDA, the content of GSH, the activities of antioxidant enzymes (CAT and Mn-SOD), and the rate of senescence and apoptosis in BMSCs [42–44]. Under oxidative stress conditions simulated by H_2_O_2_, intracellular ROS and MDA levels significantly increased (Figure 3A–C), CAT and SOD activity decreased (Figure 3D,E), and GSH content was reduced in BMSCs (Figure 3F). However, when NMNAT3 was overexpressed in BMSCs, the levels of ROS and MDA, which are indicators of intracellular oxidative damage, were reduced, CAT and SOD activity increased, and GSH content was increased (Figure 3A–F). In addition, we also found that under oxidative stress conditions, the activity of SA-β-Gal, which is related to senescence, in BMSCs overexpressing NMNAT3 was significantly reduced, the ratio of SA-β-Gal-positive cells decreased (Figure 3G,H), and apoptosis rate was significantly reduced (Figure 3I,J). Compared with the empty virus (BMSCs/Lv+H_2_O_2_) group, the difference was statistically significant (*P*<0.05). The above results showed that the antioxidative stress capacity of BMSCs modified by NMNAT3 was enhanced.

### The role of Sirt3 in mitochondrial function and antioxidative stress capacity of BMSCs overexpressing NMNAT3

Overexpression of NMNAT3 can increase NAD^+^ synthesis, improve mitochondrial function, and enhance the antioxidative stress capacity of BMSCs. Sirt3 is an important enzyme that regulates metabolism and oxidative homeostasis in mitochondria, and its catalytic activity is strongly associated with NAD^+^ levels [26,45,46]. Therefore, we further studied the role of Sirt3 in protecting mitochondrial function and enhancing antioxidative stress capacity in BMSCs overexpressing by NMNAT3. We added 3-(^1^H-1,2,3-triazol-4-yl) pyridine (3-TYP, 50 μM), a selective Sirt3 inhibitor [47], to the culture system that overexpressed NMNAT3 to inhibit Sirt3 activity, and then treated the BMSCs with 600 μM H_2_O_2_ for 24 h. The results showed that compared with the NMNAT3 overexpression (BMSCs/NMNAT3+H_2_O_2_) group, the ultrastructure of BMSC mitochondria was damaged (Figure 2A,B), the level of ATP decreased (Figure 2C), mitochondrial biogenesis regulatory factors PGC-1α and NRF1 were down-regulated (Figure 2E–G, Supplementary Figure S2), mitochondrial membrane potential decreased (Figure 2H,I), and mitochondrial function was significantly damaged in the inhibitor (BMSCs/NMNAT3+3-TYP+H_2_O_2_) group. In addition, intracellular ROS and MDA levels increased (Figure 3A–C), the activities of CAT and SOD decreased (Figure 3D,E), GSH content was reduced (Figure 3F), and cell senescence and apoptosis rates significantly increased (Figure 3G–J). These results indicate that overexpression of NMNAT3 increases NAD^+^ synthesis, which may regulate mitochondrial function and antioxidative stress capacity of BMSCs via Sirt3.

### Overexpression of NMNAT3 in BMSC reduces the acetylation level of mitochondria under oxidative stress via Sirt3

**Figure 4:**
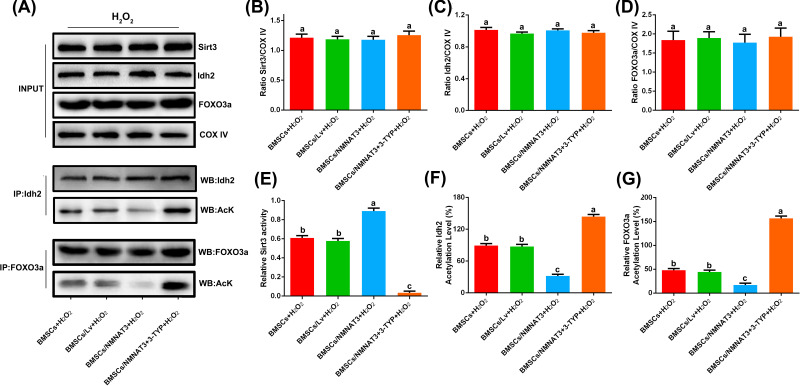
NMNAT3 enhanced the activity of Sirt3 under oxidative stress (**A–D**) Western blot analysis of Sirt3, Idh2, and FOXO3a levels in BMSCs. Acetylated Idh2 and FOXO3a were isolated by immunoprecipitation with anti-Idh2 and anti-FOXO3a antibody followed by Western blotting with anti-acetyl-lysine antibody (*n*=3). (**E**) Detection of Sirt3 relative activity in BMSCs (*n*=3), significant differences (*P*<0.05) were observed among a–c. (**F,G**) Detection of Idh2 and FOXO3a acetylation level in BMSCs, significant differences (*P*<0.05) were observed among a–c. All the experiments were repeated at least thrice (*n*≥3). Data were presented as means ± SD. *P*<0.05 by one-way ANOVA with Tukey’s post hoc test (three or more groups).

Sirt3 is mainly located in the mitochondria and plays an important role in maintaining the level of mitochondrial acetylation. Idh2 and FOXO3a are important regulatory enzymes in the mitochondria that regulate mitochondrial redox balance and maintain mitochondrial function [23,24]. The activity of these enzymes depends on the deacetylation of Sirt3. This study has determined that the expression of Sirt3, Idh2, and FOXO3a did not significantly change under oxidative stress (Figure 4A–D, Supplementary Figure S3). However, Sirt3 activity significantly decreased, and Idh2 and FOXO3a showed high acetylation rates under oxidative stress (Figure 4A,E–G, Supplementary Figure S3). However, compared with the empty virus (BMSCs/Lv+H_2_O_2_) group, the up-regulated expression of NMNAT3 significantly increased Sirt3 activity and decreased the acetylation levels of Idh2 and FOXO3a in BMSCs (Figure 4A,E–G). We further used 3-TYP to inhibit the activity of Sirt3, and the results showed that in the inhibitor (BMSCs/NMNAT3+3-TYP+H_2_O_2_) group, the acetylation level of Idh2 and FOXO3a in BMSCs modified by NMNAT3 had increased, and the effect of NMNAT3 was completely blocked (Figure 4F,G). Here, our results further showed that overexpression of NMNAT3 reduces the level of mitochondrial acetylation via the NAD^+^-Sirt3 axis, thereby improving mitochondrial function and enhancing the antioxidative stress capacity of BMSCs.

## Discussion

The efficacy of BMSCs transplantation *in vivo* is closely related to its transplant survival rate. The oxidative stress microenvironment in the transplantation area often leads to BMSCs mitochondrial dysfunction and stress apoptosis. Improving the mitochondrial function of BMSCs under stress may help improve antioxidative stress capacity and survival of BMSCs [48,49]. Many studies have shown that a reduction in intracellular NAD^+^ levels can inhibit NAD^+^-dependent enzymes activity during oxidative phosphorylation, TCA cycle and glycolysis, reduce the production of ATP, and damage the structure and function of mitochondria [17,50,51]. The reduction in NAD^+^ levels also disrupts the cellular signal molecular pathways, leading to cell senescence and apoptosis [14,52]. Therefore, maintaining a NAD^+^ horizontal steady state is an effective strategy for improving mitochondrial function and improving cell survival [15,53]. Studies have shown that NMNAT3 overexpression is an ideal method for increasing NAD^+^ levels in cells [33]. In this study, we also found that the level of NAD^+^ in BMSCs decreased significantly under oxidative stress, while the overexpression of *NMNAT3* gene in BMSCs could increase intracellular NAD^+^ levels under oxidative stress. It also effectively ameliorated the mitochondrial structural of BMSCs under oxidative stress, increased mitochondrial membrane potential, increased ATP synthesis, and promoted the expression of mitochondrial biogenesis regulatory factors (PGC-1α and NRF1) (Figure 2). Compared with the control group, the mitochondrial structure and function of BMSCs modified by NMNAT3 significantly improved, indicating that under oxidative stress, overexpression of NMNAT3 improves the mitochondrial structure and function by restoring NAD^+^ levels in cells. In addition, the activities of antioxidant enzymes (CAT and Mn-SOD) in BMSCs modified by NMNAT3 were significantly higher than in the control group, and the content of MDA and ROS significantly decreased (Figure 3), thereby delaying cell senescence and reducing apoptosis. These results can be attributed to the improvement of antioxidative stress capacity of BMSCs.

Here, we also provide the molecular mechanism of how NMNAT3 regulates mitochondrial function. In recent years, the deacetylase sirtuin protein family has been extensively investigated [47,54–56], in which Sirt3 plays an indispensable role in regulating mitochondrial energy metabolism and oxidative stress [45,46,57–59]. The biological activity of Sirt3 depends on the levels of NAD^+^. A reduction in NAD^+^ levels affects Sirt3 deacetylation, which lead to high rates of acetylation of important regulatory proteins (Idh2 and FOXO3a) in the mitochondria and ultimately dysregulation of mitochondrial function [26,28,60–63]. In this study, we detected the expression of Sirt3 and its target proteins (Idh2 and FOXO3a) under oxidative stress and found that the expression levels of Sirt3, Idh2, or FOXO3a did not significantly differ among groups, which indicated that overexpression of NMNAT3 had no effect on the translational regulation of related proteins in BMSCs.

However, we increased the level of NAD^+^ by overexpressing NMNAT3 in BMSCs and found that it could significantly increase the Sirt3 activity and significantly decrease the acetylation level of Sirt3-dependent deacetylation-related proteins (Idh2 and FOXO3a) in the mitochondria (Figure 4). This shows that NMNAT3 may increase Sirt3 activity by increasing NAD^+^ levels that then improve the function of mitochondria under oxidative stress. To further prove that overexpression of NMNAT3 regulates mitochondrial function and antioxidative stress capacity of BMSCs through Sirt3, we added an Sirt3 inhibitor after the overexpression of NMNAT3 and found that the effect of NMNAT3 was completely blocked. The acetylation levels of Idh2 and FOXO3a increased, damage to mitochondrial structure and function was aggravated, the antioxidative stress capacity of BMSCs decreased, and the rate of cell senescence and apoptosis significantly increased (Figures 2-4). Our results support that the NMNAT3-NAD^+^-Sirt3 axis is a potential mechanism to improve mitochondrial function and enhance antioxidative stress capacity of BMSCs.

## Conclusions

**Figure 5:**
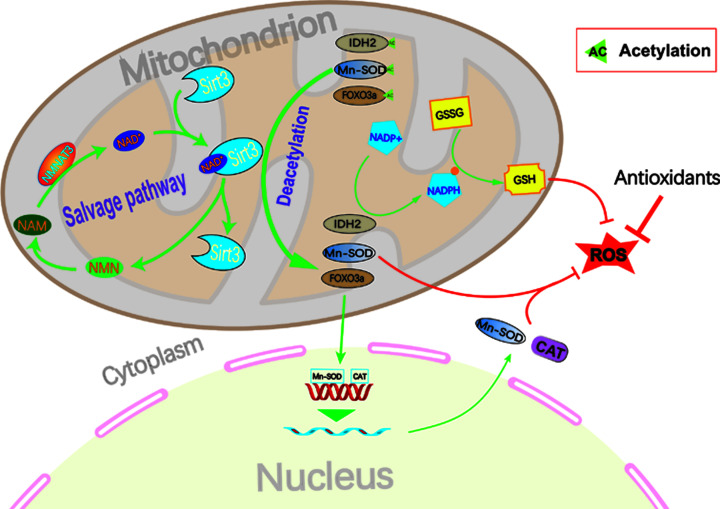
Hypothetical model of NMNAT3 regulating NAD^+^ in BMSCs to improve mitochondrial function and inhibit cell stress-induced apoptosis Under oxidative stress environment, mitochondrial function was impaired and excessive ROS production occurred, which further incurred damage to the mitochondria, leading to stress-induced apoptosis. Moreover, mitochondrial dysfunction leads to a decrease in NAD^+^ levels, which inhibits mitochondrial energy synthesis and Sirt3-mediated deacetylation of related proteins. Overexpression of NMNAT3 increases NAD^+^ remedial synthesis, promotes Sirt3-mediated deacetylation of mitochondria-related proteins, and increases mitochondrial energy synthesis and activation of antioxidant enzymes. Finally, it can improve the function of mitochondria and eliminate excessive ROS production, thereby inhibiting cellular stress-induced apoptosis.

We have demonstrated that the *NMNAT3* gene has a positive effect on improving mitochondrial function in BMSCs under oxidative stress and enhancing the antioxidative stress capacity of BMSCs using *in vitro* cell experiments, as well as proved that the NMNAT3-NAD^+^-Sirt3 axis is the potential mechanism for improving mitochondrial functions (Figure 5). The present study provides a new strategy for solving the problem of stem cell survival under oxidative stress and may be utilized in stem cell therapy.

## Supplementary Material

Supplementary Figures S1-S3

## Data Availability

The datasets during and/or analyzed during the current study available from the corresponding author on reasonable request.

## Abbreviations

ALP: alkaline phosphatase
ATP: adenosine triphosphate
BMSC: bone marrow mesenchymal stem cell
CAT: catalase
CCK-8: cell counting kit-8
CD: cluster designation of monoclonal antibodies (clusters of differentiation)
CDS: coding DNA sequence
EDTA: ethylenediaminetetraacetic acid
EP tube: eppendorf tube
FOXO3a: Forkhead-box protein O3a
H_2_O_2_: hydrogen peroxide
Idh2: isocitrate dehydrogenase 2
l-DMEM: l-glutamine Dulbecco’s modified Eagle’s medium
Lv: lentiviral vector
MDA: malondialdehyde
Mn-SOD: manganese superoxide dismutase
MOI: multiplicity of infection
NAD^+^: nicotinamide adenine dinucleotide
NMNAT3: nicotinamide mononucleotide adenylyl transferase 3
NRF1: nuclear respiratory factor 1
PBS: phosphate-buffered saline
PGC-1α: peroxisome proliferator-activated receptor γ coactivator-1α
qPCR: quantitative real-time PCR
ROS: reactive oxygen species
SA-β-gal: senescence β-galactosidase
Sirt3: silent mating type information regulation 2 homolog 3
TCA: tricarboxylic acid
TMRE: tetramethylrhodamine ethyl ester
3-TYP: 3-(^1^H-1,2,3-triazol-4-yl) pyridine
