# Supplementary material for: Overexpression of NMNAT3 improves mitochondrial function and enhances antioxidative stress capacity of bone marrow mesenchymal stem cells via the NAD+-Sirt3 pathway
Source: Biosci Rep. 2022 Jan 14;42(1):BSR20211005. doi: 10.1042/BSR20211005 (PMC8762348; doi:10.1042/BSR20211005)
Supplement: Supplementary Figures S1-S3 [file BSR-2021-1005_supp.pdf]

Supplementary Files

Supplementary Figure 1

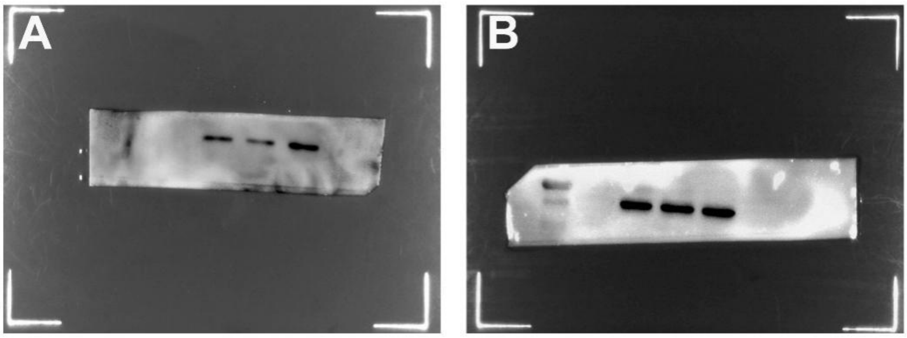

Supplementary figure from Figure 1B in the main article: Western blot analysis of NMNAT3 protein expression**. (A)** NMNAT3 protein band detected by Western blots; **(B)**β-actin protein band detected by Western blots. Grouped from left to right: control group, Lv-EGFP, and Lv-NMNAT3.

Supplementary Figure 2

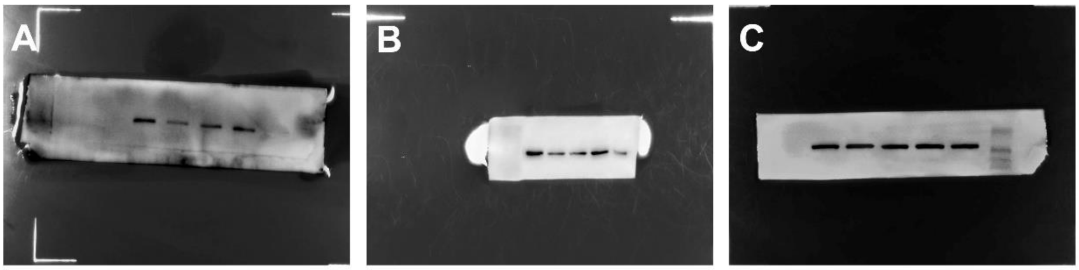

Supplementary figure from Figure 2F in the main article: Western blot analysis of PGC-1α and NRF1 expression in mitochondria. **(A)** PGC-1α protein band detected by Western blots; **(B)** NRF1 protein band detected by Western blots; **(C)** COX IV protein band detected by Western blots. Grouped from left to right: BMSCs, BMSCs+H_2O_2,

BMSCs/Lv +H_2O_2, BMSCs/NMNAT3+H_2O_2, and BMSCs/NMNAT3+3-TYP+H_2O_2.

Supplementary Figure 3

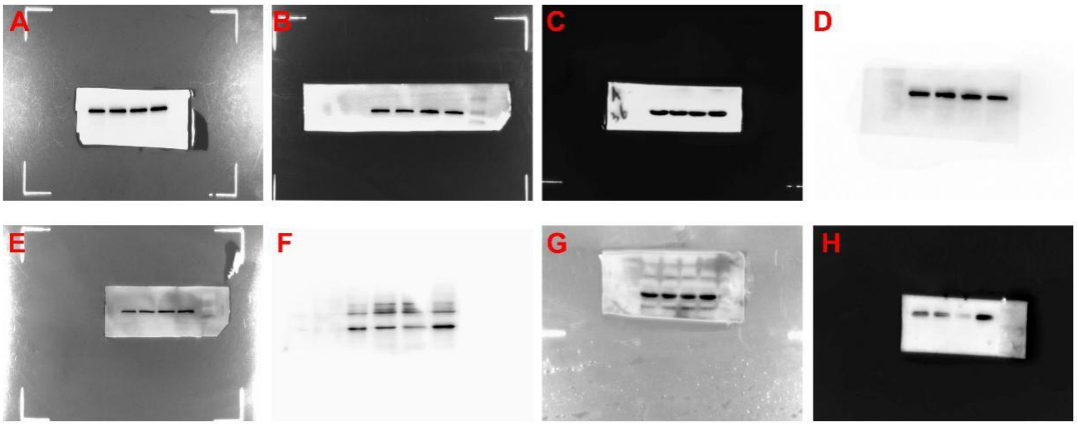

Supplementary figure from Figure 4A in the main article: Western blot analysis of Sirt3, Idh2 and FOXO3a levels in BMSCs, Acetylated Idh2 and FOXO3a were isolated by immunoprecipitation with anti-Idh2 and anti-FOXO3a antibody followed by western blotting with anti-acetyl-lysine antibody. **(A)** Sirt3 protein band detected by Western blots; **(B)** Idh2 protein band detected by Western blots; **(C)** FOXO3a protein band detected by Western blots; **(D)** COX IV protein band detected by Western blots; **(E)** Idh2 protein band detected by Western blots with anti-Idh2 antibody; **(F)** Acetylated Idh2 protein band detected by Western blots with anti-acetyl-lysine antibody; **(G)** FOXO3a protein band detected by Western blots with anti- FOXO3a antibody; **(H)** Acetylated FOXO3a protein band detected by Western blots with anti-acetyl-lysine antibody. Grouped from left to right: BMSCs+H_2O_2, BMSCs/Lv +H_2O_2, BMSCs/NMNAT3+H_2O_2, and BMSCs/NMNAT3+3-TYP+H_2O_2.
